# A single nucleotide substitution introducing premature stop codon within *CsTFL1* explains the *determinate-2* phenotype in cucumber (*Cucumis sativus* L.)

**DOI:** 10.1038/s41598-024-76549-w

**Published:** 2024-10-25

**Authors:** Bartosz Biernacik, Renata Słomnicka, Karolina Kaźmińska, Szymon Mużacz, Grzegorz Bartoszewski

**Affiliations:** Department of Plant Genetics Breeding and Biotechnology, Institute of Biology, Warsaw University of Life Sciences, Warsaw, Poland

**Keywords:** Cucurbits, Determinate growth, Plant architecture, *TFL1*, SAM transition, Plant breeding, Agricultural genetics

## Abstract

The determinate growth habit of plants reduces the number of internodes and shortens the main stem by terminating the shoot apical meristem through a transition to inflorescence. Understanding the genetic basis of this habit can help optimize crop yield and cultivation technology for vegetable breeding. This study aimed to identify the *determinate-2* (*de-2*) gene responsible for the determinate growth habit in the W-sk cucumber line. Termination of the main stem in the W-sk line occurred between 14 and 23 internodes, depending on cultivation conditions. Resequencing of the W-sk genome identified a novel SNP in the cucumber *TERMINAL FLOWER1* (*CsTFL1*) gene, explaining the *de-2* phenotype. This was verified with a CAPS-T marker cosegregation with determinate growth in the F_2:3_ population, and this polymorphism is unique among genotyped indeterminate cucumber cultivars or breeding lines. Crossing the W-sk line with the G421 line with the *determinate* (*de*) gene confirmed the allelism of both genes. An SNP in *CsTFL1* in the W-sk line introduced a premature stop codon, resulting in the putative deletion of 13 amino acids, possibly causing determinate growth habit. Overall, this study provides insights into the genetic basis of cucumber plant growth architecture and advances in cucumber breeding.

**Supplementary Information:**

The online version contains supplementary material available at 10.1038/s41598-024-76549-w.

## Background

The main growth habits of most flowering plants are indeterminate and determinate. Plants exhibiting indeterminate growth have infinite potential for growth of the main stem, because their shoot apical meristem (SAM) or inflorescence meristem (IM) are maintained in an indeterminate state and new internodes can develop. In plants characterized by determinate growth, the main stem is terminated by the transition of the SAM to the IM, from which flowers develop^1^. Some cultivars of indeterminate growth crops exhibit determinate growth habit, such as tomato (*Solanum lycopersicum* L.), soybean (*Glycine max* L.), sesame (*Sesamum indicum* L.), faba beans (*Vicia faba* L.), and cucumber (*Cucumis sativus *L.)^2–6^. In many crops, determinate cultivars are more advantageous because of the shorter length of the main stem, denser fruit distribution, and synchronous growth and ripening of fruits, which can benefit mechanical fruit harvest. Modern crop breeding should consider the molecular basis of determinate growth, which is strongly related to the mechanism of SAM transition into IM^7,8^.

The transition of SAM to IM is initiated by florigen, which was described by Chailakhyan in 1937^9^. Florigen was identified as a mobile FLOWERING LOCUS T (FT) protein produced in leaves and transported to the SAM, where it interacts with the bZIP transcription factor FLOWERING LOCUS D (FD) or FD PARALOG (FDP), triggering the expression of floral transcription factor APETALA1 (AP1) and further expression of LEAFY (LFY), determining inflorescence identity^10–13^. The TERMINAL FLOWER1 (TFL1) protein is produced in SAM and is a major inhibitor competing with FT to keep the meristem in a state of indeterminancy^14,15^. In rice, the FT homolog HEADING DATE 3 A (Hd3a) interacts with 14-3-3 proteins to be imported to the nucleus and binds with FD to form the floral activation complex (FAC) to regulate expression of *AP1*/*LFY*^16^. This interaction was confirmed in *Arabidopsis*, where a similar complex has been described^17^.

Both the FT and TFL1 proteins are members of the PHOSPHATIDYLETHANOLAMINE BINDING PROTEINs (PEBPs), a family of proteins with high affinity for phospholipids. In plants, a small family of genes encoding PEBPs exists; for example, in cucumber, eight genes encoding PEBPs have been identified^18^. Well-known members of the PEBPs family are CENTRORADIALIS (CEN), TERMINAL FLOWER1 (TFL1), and SELF-PRUNING (SP), all of which are involved in the SAM transition to the IM. Mutations in *Antirrhinum* CEN result in terminal flower development and termination of the inflorescence^19^. *Antirrhinum* TFL1 mutants exhibit terminal flowers, and TFL1-overexpressing plants flower late and develop additional lateral shoots lacking subtending cauline leaves instead of flowers^20^. Mutation in tomato SP results in the termination of main stem sympodial growth^21,22^. Despite its central role in growth architecture, the regulatory network of TFL1 in crop species is still insufficiently understood^23,24^.

The determinate growth habit of cucumber plants has attracted the attention of researchers and breeders for years, and the genetic basis of this growth habit has been studied. Hutchins reported that a single dominant gene determines growth in cucumber^25^. Later, other determinate growth genes were reported as single, recessive genes^26–28^. The allelism tests were conducted with lines containing each of the mentioned determinate growth genes and concluded that all of them are located in the same locus assigned as *determinate* (*de*), but their expression is dependent on environmental conditions and genetic background^29^. Further studies have resulted in mapping of the *de* locus on chromosome 6, and the ortholog of *TFL1* was suggested as a candidate for *de*^30–33^. Based on fine mapping and functional studies, *CsTFL1* (Csa6G452100) was identified as a gene responsible for determinate growth^34^. Detailed studies of the G421 line, characterized by determinate growth, revealed a nonsynonymous single nucleotide polymorphism (SNP), T-to-C, in the first exon of *CsTFL1*, resulting in the substitution of serine 71 with proline (S71P) and explaining determinate growth. Downregulation of *CsTFL1* in cucumber resulted in determinate growth, and ectopic overexpression of *CsTFL1* in *Arabidopsis* resulted in delayed flowering of the wild type and *tfl1-11* mutant phenotype rescue, confirming that *CsTFL1*is responsible for determinate growth. Analysis of the CsTFL1 regulatory network revealed that CsTFL1 interacts through the cucumber homolog of NEGATIVE ON TATA LESS2 (CsNOT2a) with CsFDP to regulate SAM transition in cucumber^34^. Studies of the cucumber line WI1983Hde, characterized by a severe determinate growth habit, revealed deletion of a 20-kb genomic region, including the *CsTFL1* gene, which also showed that another gene is involved in SAM transition in cucumber^35^. Studies on lines D226 and D082 revealed a *determinate-novel (det-novel)* locus conditioning growth habit, and a candidate gene, *CsCEN* (Csa6G152360), a homolog of *CsTFL1* also known as *CsTFL1d*, was identified. Silencing of *CsCEN/CsTFL1d* in cucumber resulted in determinate growth and extreme decreases in plant height and the number of nodes^36^. Studies have also revealed that the determinate growth of cucumber plants, conditioned by *CsTFL1* or *CsCEN/CsTFL1d*, can be partially rescued by environmental factors such as long days or low and high temperatures^27,35,36^. Another *CsTFL1* homolog, *CsTFL1b*, was also linked with control of main stem termination, but ectopic expression of this gene in* Arabidopsis tfl1* mutant did not restore wild-type phenotype completely. Although, ectopic expression of *CsTFL1b* in wild type *Arabidopsis* caused delayed bolting and flowering with flowers that could transite to new inflorescence shoots^33^. Also, it was suggested that ethylene production can influence determinate growth habit in cucumber^37^.

Another recessive gene, conditioning determinate growth in cucumber, *determinate-2* (*de-2*), has been described in the W-sk line^38^. This line was developed as a result of ethylene imine mutagenesis of an inbred line selected from the cucumber cultivar Borszczagowski^39^. To date, no candidate gene for *de-2* has been proposed. The aim of this study was to identify the *de-2* gene responsible for determine growth habit of the W-sk line. To identify this gene, the inbred line B10 was crossed with W-sk to develop a segregating population, and the genomes of the parental lines were resequenced. Molecular marker was designed for SNP in gene candidate and used for genotyping of the segregating population and cucumber cultivars and breeding lines. In this study, we demonstrated that the determinate growth of the cucumber W-sk line is affected by a novel mutation in the *CsTFL1* gene.

## Materials and methods

### Plant materials

The cucumber line W-sk was developed by self-pollination (> S10) of a single plant characterized by determinate growth identified in the mutagenesis experiment of an inbred line developed from the cultivar Borszczagowski^38,39^. The monoecious cucumber inbred line B10, which was used for mutagenesis and subsequently self-pollinated (> S15), and the gynoecious line Gy14 served as controls. For the allelism test, the gynoecious line G421, characterized by determinate growth conditioned by the *determinate (de)*gene, as described by Wen et al^34^., was used. Seeds of all plants were sowed in multi-cell trays with peat mix in a plastic tunnel, and seedlings were planted directly into the soil for open field or plastic tunnel experiments or pots for greenhouse experiments. The plants in the plastic tunnel and greenhouse grew by the strings and were watered daily. All these lines were maintained at the Wolica Experimental Station at the Department of Plant Genetics Breeding and Biotechnology, Warsaw University of Life Sciences (DPGBB-WULS, Warsaw, Poland).

### Phenotyping of the W-sk line

The growth habit and morphological traits of the W-sk line were evaluated in comparison to those of the B10 control line by phenotyping eight-week-old plants in the field and plastic tunnel in the summer of 2021. Seeds were sowed in a plastic tunnel on May 22. 2021, and seedlings were planted in the field and plastic tunnel in summer on June 7. 2021. The average temperature at the field for the cultivation period was 21 ℃; in the plastic tunnel, it was 4–5 ℃ higher. The length of the main shoot, number and length of main shoot internodes, number and length of lateral shoots, width and length of the leaf blades, length of the leaf petioles, length diameter and weight of the mature fruits were assessed for 6–18 plants for each line.

### Genetic analysis and segregating population development

The indeterminate lines B10 and Gy14 were crossed with the determinate line W-sk carrying the *de-2* gene to developed families of F_1_, F_2_, BC_1_ (backcross to B10) and BP_2_ (backcross to W-sk) and investigate the inheritance of *de-2* gene. Both populations were grown under plastic tunnel conditions in May-June 2022 at the Wolica Experimental Station of DPGBB, WULS (Warsaw, Poland). The growth habit of each plant (determinate or indeterminate) was described at the flowering eight-week-old plant stage. Plants with the phenotype of W-sk (termination of the main stem with a cluster of flowers at the top, reduced lateral brunches) were classified as determinate. Wild-type F_2_ individuals from B10 × W-sk were self-pollinated to obtain F_3_ seeds for genetic evaluation of F_2_ individuals. The plant growth habits of the B10 × W-sk F_3_ families were evaluated in a field experiment performed in May–June 2023. Seedlings of F_3_ families were planted in May in a plastic tunnel and transplanted to the field in July 2023. The growth habit of 16–24 plants of each F_3_ family was evaluated by visual inspection.

### Allelism test

Line G421 carrying the *de* gene was crossed as a female parent with the W-sk line carrying *de-2* as a male, and F_1_ seeds were produced. On March 8. 2024, G421, W-sk, and F_1_ seeds were planted in a greenhouse in 15-liter pots filled with Klasmann TS1 peat mix (Klasmann-Deilmann, Geeste, Germany) and cultivated with the temperature at the day 25 ℃ and 22 ℃ at night. The growth habit of eight-week-old plants were evaluated, and the number of internodes was counted.

### Statistical analysis

The means of the measurements and counts of three replicates for W-sk and B10 plants were statistically analyzed. GraphPad Prism v8.0 (GraphPad Software Inc., San Diego, CA, USA) was used for basic statistics such as the mean and standard deviation (SD) among the lines. Statistica 12 software (TIBCO Software Inc., Palo Alto, CA, USA) was used to perform a Student’s t-test for statistical analysis of the phenotyping data, and the segregation of developed families was statistically analyzed with the *Chi*^*2*^ test with a significance level of *p* = 0.05.

### DNA isolation and genome resequencing

Total genomic DNA was extracted from the leaves of young plants grown in a plastic tunnel as described previously^40^. DNA was isolated using a NucleoSpin Plant II Kit (Macherey-Nagel, Düren, Germany) according to the manufacturer’s instructions. The genomes of the B10 and W-sk lines, which were used for developing the F_2:3_ segregating population, were resequenced using the Illumina NovaSeq 6000 platform (Novogene, Cambridge, UK) (Supplementary Table S1). Sequencing data were filtered and aligned with the cucumber reference genome B10v3^41^using BWA, and single nucleotide polymorphisms (SNPs) and insertion‒deletions (InDels) variant calling analysis were performed with SAMtools^42,43^. The genomic region corresponding to the *CsTFL1*gene was analyzed using genomic data available at the Cucurbit Genomics Database v2—CuGenDBv2^44^, accessed on April, 2024.

### CAPS marker development and genotyping

Primers for PCR were designed using Oligo7 software (Molecular Biology Insights Inc., Cascade, CO, USA) and synthesized by Genomed SA (Warsaw, Poland) (Supplementary Table S2). PCR was performed using DreamTaq DNA Polymerase according to the protocol provided by the enzyme supplier (Thermo Fischer Scientific, Waltham, MA, USA). The amplicon TFL1-4 was subjected to Sanger sequencing to verify the SNP identified in exon 4 of *CsTFL1*. The cleaved amplified polymorphic sequence marker CAPS-T was developed for the A/T SNP in *CsTFL1*. The digestion of the CAPS amplicon was conducted at 65 °C for 16 h using the TasI enzyme (Thermo Fischer Scientific, Cleveland, OH, USA). All reactions and restriction digests were performed using a PTC-200 thermal cycler (Bio-Rad, Hercules, CA, USA). Amplicons and restriction fragments were visualized using agarose gel electrophoresis and ethidium bromide DNA staining. The F_2:3_ segregating population was genotyped with the CAPS-T marker. In total, 178 F_2_ plants were used for genotyping. The CAPS-T marker was also tested on a set of 63 cucumber cultivars and inbred lines.

### RNA isolation and RT-qPCR

Shoot apices were collected from seven-week-old plants grown in June-July 2021 in a plastic tunnel and stored in liquid nitrogen. Tissues were collected from F_2_ wilt-type homozygous plants and *determinate-2* homozygous plants selected by growth-type phenotyping and genotyping with CAPS-T marker. Three pools of tissues representing three wild-type plants and three pools representing three *determinate-2* plants were composed. Each pool’s tissue was homogenized with mortar and pestle, and RNA was isolated with RNeasy Plant Mini Kit (Qiagen, Hilden, Germany). RNA was treated with DNase I (EURx, Gdańsk, Poland), and cDNA synthesis was performed with a High-Capacity cDNA Reverse Transcription Kit (Applied Biosystems, Waltham, MT, USA). RT-qPCR analysis was performed for three biological replicates, one pool as replicate, and three technical replicates as described by Mróz et al.^45^. Primers are provided in Supplementary Table S2. Relative expression of *CsTFL1* was assessed using three cucumber reference genes, *TIP41*,* UBI-ep*, and *CACS*. Calculations were done with the 2^−ΔΔCt^method^46^.

### Bioinformatic analysis of CsTFL1

Aligned reads from the resequencing experiment were viewed with IGV^47^. The amino acid sequence prediction of the truncated protein from W-sk was made with Translate tool from Expasy (https://web.expasy.org/translate/). Alignment of protein sequences was made with Omega Clustal software^48^with sequences retrieved from UniProt^49^, NCBI GenBank^50^, and CuGenDBv2^44^databases. Conserved regions in the protein alignment were defined based on the previous studies^51,52^. All sequences are listed in Supplemental Table S3. Protein structure prediction was done in Colab Notebook with AlphaFold2 (ColabFold)^53^, and images were captured with PyMOL v3^54^.

## Results

### Phenotypic characterization of the W-sk line

**Fig. 1:**
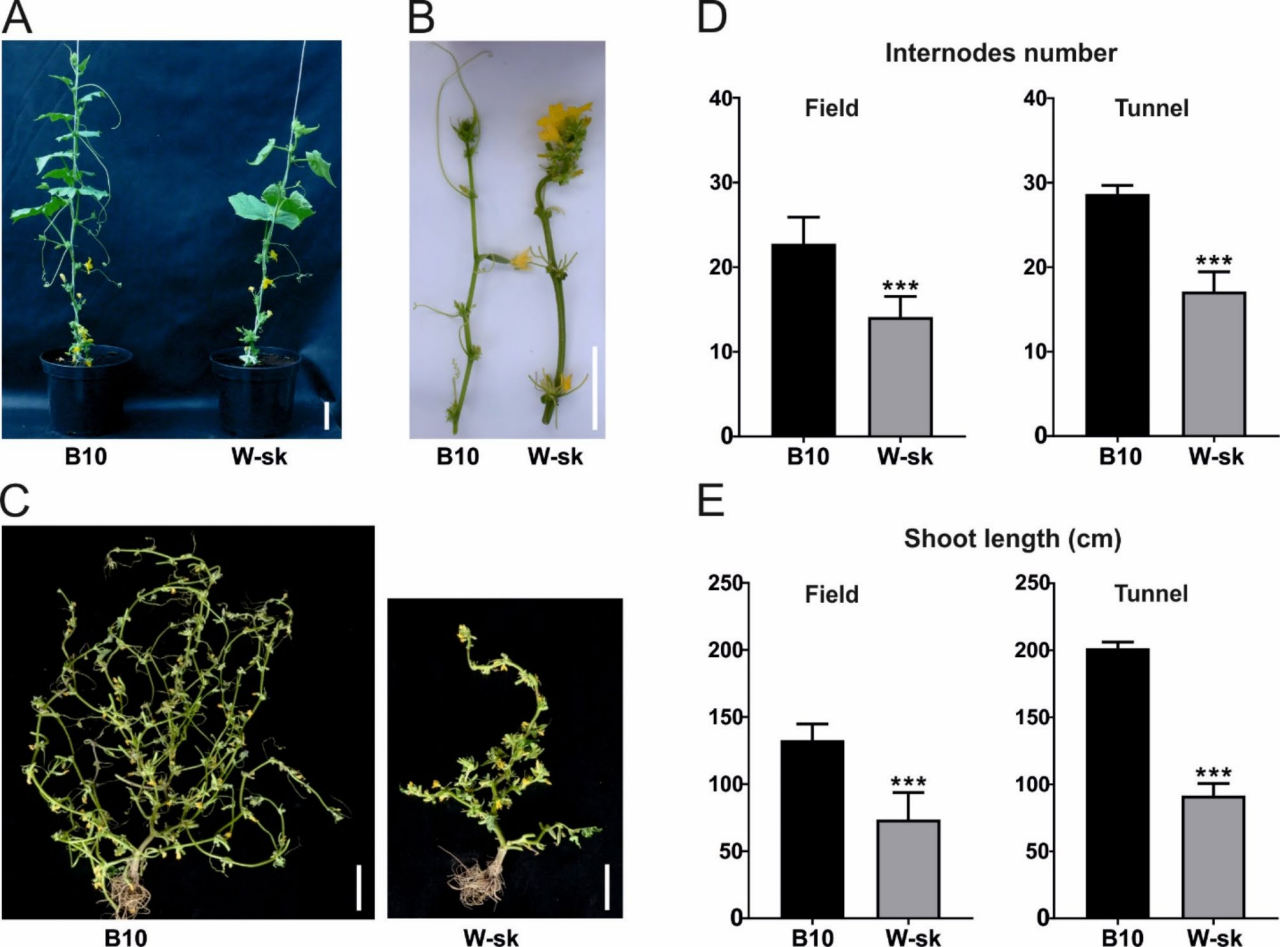
Phenotype of the determinate growth W-sk line carrying the *de-2* gene and the indeterminate control line B10. **A** Plant architecture of B10 and W-sk (leaves and lateral shoots from the first 10 nodes were removed). **B** Main shoot apical part of B10 and W-sk. **C** Main and lateral shoots of 8-week-old plants grown in the field (leaves were removed). **D** Average internode number for B10 and W-sk plants grown in the field and plastic tunnel. **E** Average length of the main shoots of B10 and W-sk plants grown in the field and plastic tunnel. The data are presented as the means ± SDs of three biological replicates with 3 plants in each replicate *** Student’s t–test *P* < 0.001. White bars represent 10 cm.

The termination of the indeterminate growth of W-sk plants carrying the *determinate-2* gene occurred in six- to seven-week-old plants, and it was characterized by developing clusters of flowers and small narrow leaves, which formed characteristic rosette-like structures at the apexes, which prevented forming new internodes terminating the growth of main stem (Fig. 1A–C). The termination of the indeterminate growth of W-sk plants occurred when plants possessed an average of 14.1 ± 2.4 internodes in the field and 17.1 ± 2.3 internodes in the plastic tunnel, while at the same time, B10 plants possessed an average of 22.8 ± 3.1 internodes in the field and 28.7 ± 1 internodes in the plastic tunnel. The length of internodes of W-sk plants was slightly shorter (Supplementary Figure S1A). Overall, in 8-week-old plants, both in the field and in the plastic tunnel, the length of the main shoots of W-sk plants was reduced by approximately half compared to B10 plants (Fig. 1D, E). W-sk plants developed 3–4 times fewer lateral shoots, which were much shorter than those of B10 plants (Supplementary Figure S1B). Compared with those of B10 plants, the leaves of eight-week-old W-sk plants were slightly longer and wider, but the lengths of the petioles did not differ (Supplementary Figure S1C). The fruits of the W-sk line grown in the field were longer with smaller diameters and lower fruit weights (Supplementary Figure S1D). Thus, the determinate growth of the W-sk line is combined with other phenotypic differences.

### Genetic analysis of determinate growth of the W-sk line

The control line B10 was crossed with W-sk, and F_1_, F_2_, and back-crossed progenies were analyzed for growth type. The plants of the F_1_ and BC_1_ generations exhibited indeterminate growth. In the F_2_ population, there were 130 indeterminate plants (*De-2/--*) and 50 determinate plants (*de-2/de-2*). Among 100 BC_2_ plants, 47 were indeterminate (*De-2/de-2*) and 53 were determinate (*de-2/de-2*). The segregation ratio for F_2_ was 3:1, and that for BC_1_P_2_ was 1:1 (Table 1). Similar results were obtained for the Gy14 × W-sk cross (Table 1). These results confirmed that a single recessive gene (*de-2*) underlies the determinate growth habit of W-sk.

**Table 1:** Genetic analysis of growth habit in parental lines and segregation populations derived from crosses of indeterminate growth lines B10 and Gy14 with the determinate growth line W-sk carrying *the determinate-2* gene.

Cross	Generation	Number of plants	Growth habit		Segregation ratio	Chi^2^ value
			Indeterminate *De-2/--*	Determinate *de-2/de-2*		
B10 × W-sk	P_1_ (B10)	10	10	0	D	–
	P_2_ (W-sk)	10	0	10	d	–
	F_1_ (B10 × W-sk)	10	10	0	D	–
	F_2_ (B10 × W-sk)	180	130	50	3:1	0.75
	BC_1_ (F_1_ × P_1_)	20	20	0	D	–
	BC_2_ (F_1_ × P_2_)	100	47	53	1:1	0.36
Gy14 × W-sk	P_1_ (Gy14)	10	10	0	D	–
	P_2_ (W-sk)	10	0	10	d	–
	F_1_ (Gy14 × W-sk)	10	10	0	D	–
	F_2_ (Gy14 × W-sk)	240	190	50	3:1	2.20
	BC_1_ (F_1_ × P_1_)	60	60	0	D	–
	BC_2_ (F_1_ × P_2_)	115	65	50	1:1	0.36

We crossed the determinate line G421 carrying *de* gene with W-sk carrying *de-2* to test these genes for allelism. All 40 F_1_ individuals exhibited determinate growth, indicating that *de-2* is a new allele in the *de* locus. In this experiment, G421 plants developed, on average, 28 internodes, whereas W-sk line 23 and F_1_ plants developed 33 internodes with a heterosis effect observed (Supplementary Figure S2).

### Single nucleotide substitution in *CsTFL1* explains the *determinate-2* phenotype

**Fig. 2:**
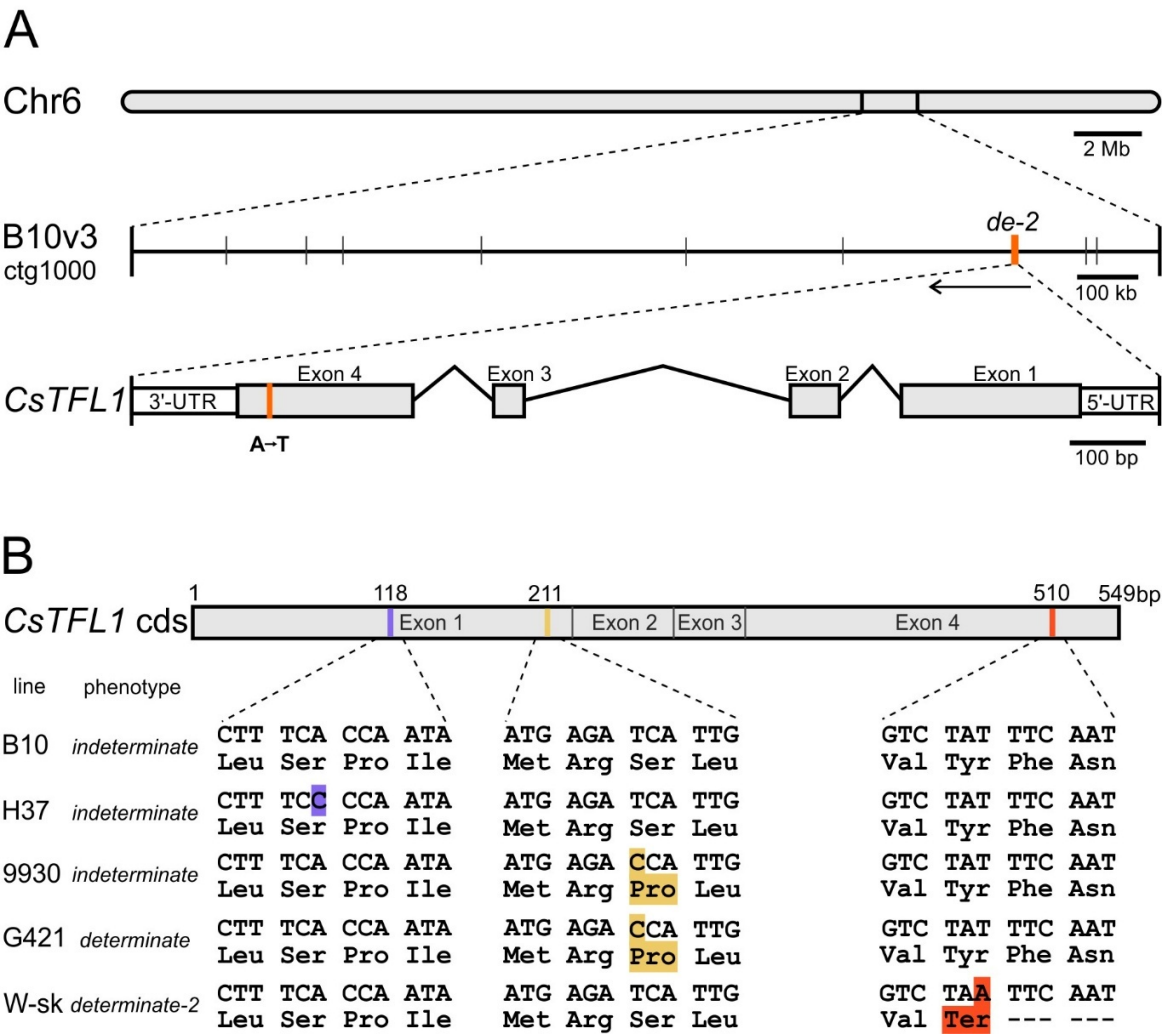
Mapping of the *determinate-2* (*de-2*) gene in the cucumber genome and changes in the amino acid sequence of CsTFL1. **A** A chromosome 6 scheme based on 9930v3 cucumber genome sequence with a region corresponding to contig ctg1000 of the B10v3 genome and a transcribed region (CDS) of *CsTFL1* (Cucsat.G1553) gene with an A to T substitution in exon 4 (orange line). The *CsTFL1* gene is located on chromosome 6 in the opposite direction. *de-2* is the *determinate-2* locus. Other SNPs detected in W-sk are marked with black vertical lines. The detailed positions of the SNPs are provided in Supplementary Table S4. **B** Single nucleotide substitutions identified in the coding regions of *CsTFL1* and protein differences in cucumber lines characterized by indeterminate (B10, H37, and 9930) and determinate (G421 and W-sk) plant growth habit. A violet synonymous A-to-C substitution has no effect on the amino acid sequence, a yellow non-synonymous T-to-C substitution results in a Ser-to-Pro substitution (S71P), and an orange non-synonymous T-to-A substitution in the W-sk line introduces a premature stop codon Tyr-to-Ter (Y170STOP).

The genomes of the parental cucumber lines were resequenced. Filtered reads were mapped on the B10v3 reference, and SNPs and InDels were called (Supplementary Table S1). As the result of the analysis, a single SNP in the *CsTFL1* gene located on contig ctg1000 (Cucsat.G1553) was identified. This A-to-T nonsynonymous substitution was located in exon 4, the last exon of *CsTFL1* (Fig. 2A). There were no other SNPs or InDels in the coding regions of the genes located on ctg1000 (Supplementary Table S4), which is a part of chromosome 6. The identified SNP in *CsTFL1* was confirmed by PCR amplification and sequencing of the exon 4 region. Based on this SNP, the molecular marker CAPS-T was developed and tested on a segregating population developed from a cross of B10 × W-sk (178 F_2_individuals). The marker completely cosegregated with the determinate plant growth habit (Supplementary Table S5). The marker was also tested on a set of 63 cucumber cultivars and breeding lines characterized by indeterminate growth, and the results were consistent with the type of growth (Supplementary Table S6). In addition, cucumber USDA core collection resequencing data^55^, deposited at CuGenDBv2, were checked for the presence of the SNP, which was not found in any of the 388 accessions. Taken together, these findings confirm that the A-to-T SNP in *CsTFL1* exon 4 explains the *determinate-2* phenotype of the W-sk line.

### Effect of SNP on *CsTFL1* expression and protein structure

**Fig. 3:**
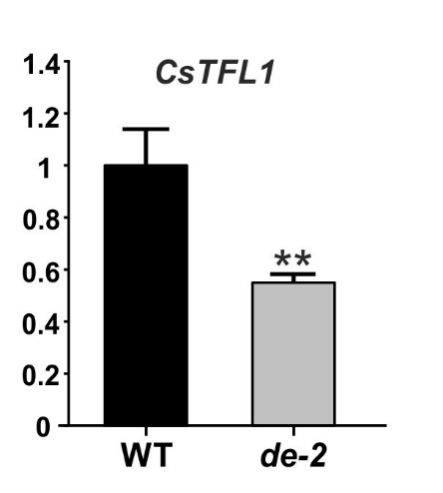
Relative expression of *CsTFL1* in *determinate-2* (*de-2*) cucumber plants compared to wild-type plants analyzed with RT-qPCR. Expression was assessed for shoot apexes from seven-week-old plants. **Student’s t–test *P* < 0.01.

**Fig. 4:**
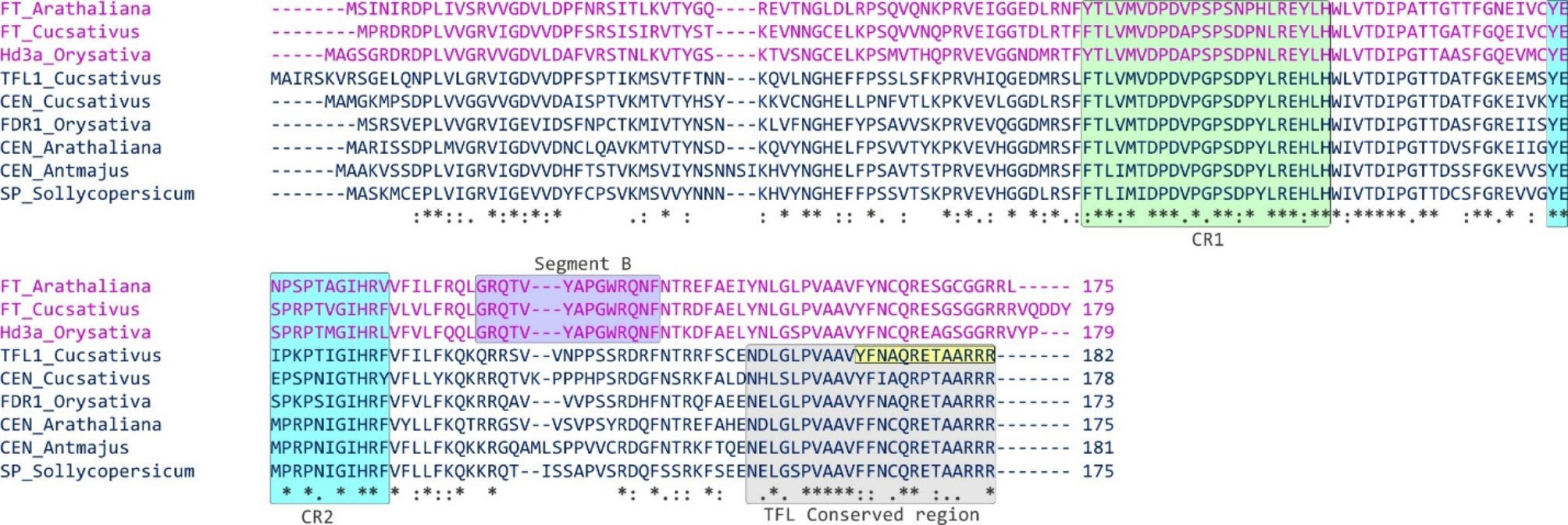
Comparison of amino acid sequences and conserved segment alignment of FT/TFL1/CEN proteins from *Cucumis sativus*,* Oryza sativa*, *Arabidopsis thaliana*, *Antirrhinum majus* and *Solanum lycopersicum*. FT proteins are shown in pink, and TFL1 are shown in dark blue. CR1 and CR2 segments are conserved in both TFL1 and FT proteins, segment B is conserved only in FT proteins. Yellow fields indicate thirteen amino acid deletion in CsTFL1 in the *determinate-2* line W-sk as a consequence of an A-to-T substitution in exon 4. The numbers and sources of sequences are provided in Supplementary Table S3.

**Fig. 5:**
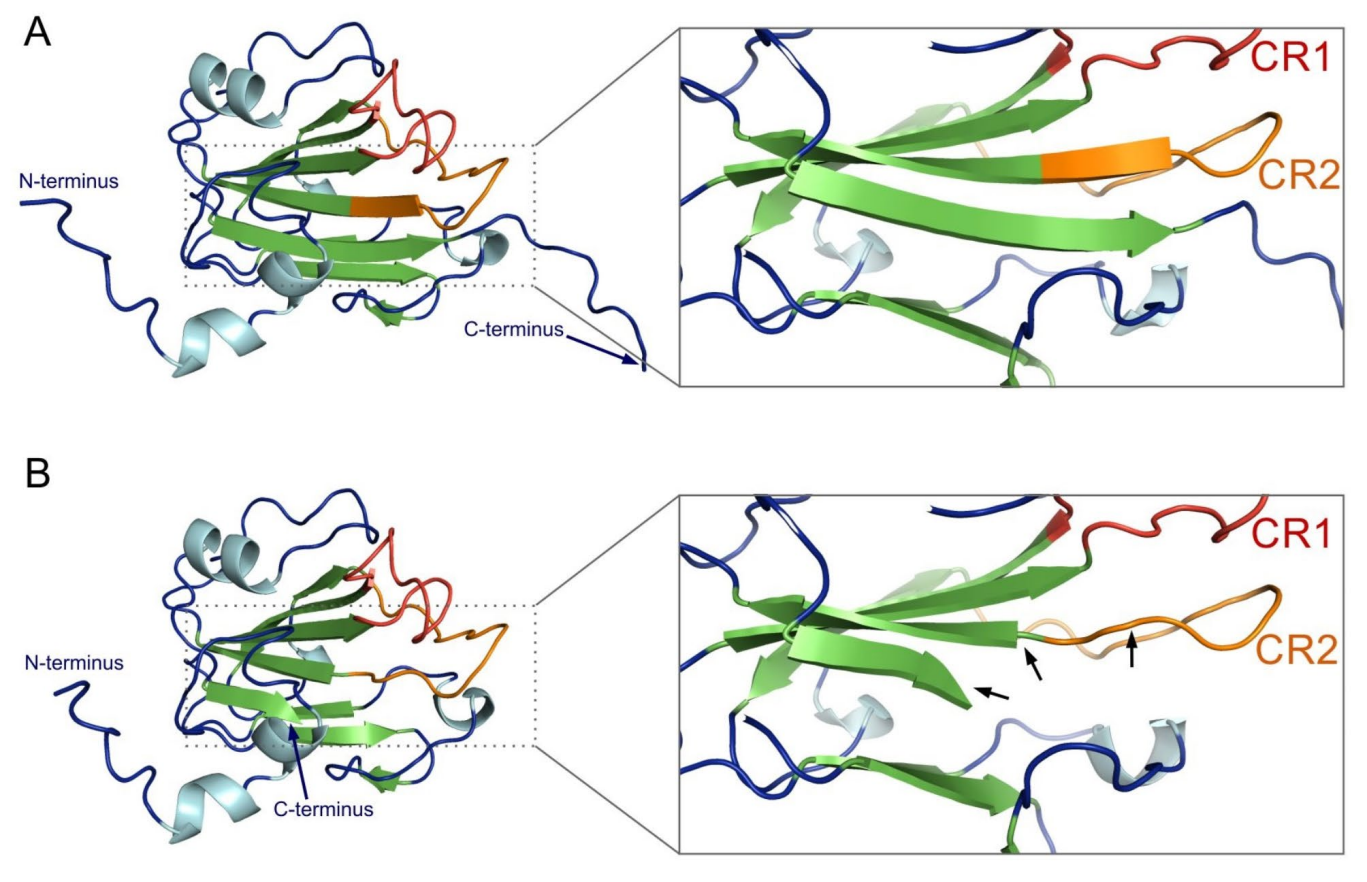
Predicted protein models of wild-type CsTFL1 from line B10 (**A**) and CsTFL1 with a premature stop codon at the C-terminus (Y170STOP) from line W-sk (**B**). Secondary structures are colored light blue for α-helices and green for β-sheets. Regions involved in the anion-binding pocket surface structure are highlighted in red (CR1) and orange (CR2). The 13 amino acid deletion results in the truncation of the last β-sheet, which alters the parallel β-sheet arrangement, as indicated by the arrows.

The *CsTFL1* expression was evaluated in shoot apexes by RT-qPCR. This analysis revealed that the expression of *CsTFL1* is reduced in *determinate-2* plants, carrying substitution in the *CsTFL1* gene (Fig. 3); however, in both types of plants, the gene was expressed. The identified A-to-T substitution in exon 4 of *CsTFL1* results in the introduction of a premature stop codon at the C-terminus of CsTFL1 (Y170STOP) (Fig. 2B). This stop codon is predicted to lead to the truncation of CsTFL1 and the deletion of 13 C-terminal amino acids (YFNAQRETAARRR) (Fig. 4), which are in the conserved region of the C-terminus of TFL1 proteins (TFL1/CEN/SP/FDR1) among different notable species. This might suggest a role of this region in the functionality of TFL1 proteins. Spatial structure prediction of CsTFL1 for B10 and W-sk was predicted to check if disruption of this conserved region could have an effect on protein structure (Fig. 5). Comparison of the CsTFL1 proteins revealed truncation in the last β-sheet being in contact with a parallel β-sheet that partakes in the structure of the anion-binding pocket (CR1, CR2), which is crucial in inhibiting or activating the role of the protein^56,57^. These might affect the function of CsTFL1 since it would change how putative ligand interacts with anion-binding pocket or how this protein folds and interacts with other proteins.

## Discussion

Plant architecture plays a role in plant evolution and crop domestication and is an important trait in crop improvement. In several crop species, the development of determinate growth cultivars has resulted in significant changes in plant production systems. Determinate cultivars are characterized by early flowering and uniform yield, allowing for high-density planting and mechanical harvesting and less labor production^58–60^. There have been attempts to develop determinate cucumber cultivars not only for open field or plastic tunnel production but also for urban farming^61,62^.

In this study, we identified a single nucleotide substitution in the *CsTFL1* gene explaining the *determinate-2 *phenotype of the cucumber W-sk line, which was developed from a chemical mutagenesis experiment^38^. The growth of the W-sk line was compared with that of the B10 line used previously for mutagenesis^39^. Since the mutagenesis experiment, both lines were subsequently self-pollinated and are now highly inbred (> S10). Termination of the main stem of the W-sk line occurred in six- to seven-week-old plants, and a characteristic rosette of male flowers and small narrow leaves that formed at the suppressed SAM was observed. In our study, line W-sk produced on average 14 internodes in the field, 17 internodes in the plastic tunnel, and 23 internodes in the greenhouse, depending on the growth conditions. Sołtysiak et al.^38^. reported 18–20 internodes for this line in field experiments, more than in our study. This difference may be related to the level of W-sk inbreeding, which is currently a highly inbred line, or environmental conditions. We observed a greater number of internodes in the plastic tunnel or greenhouse. This finding is in agreement with observations for other cucumber determinate lines, where it was demonstrated that day length, temperature, and light intensity can influence the number of internodes^27,35,36^. Based on the phenotypic observations and reports on the other determinate lines, it seems that the W-sk line can be classified as a moderate determinate line. It is not as mild as G421 with a substitution in the *CsTFL1* gene (27 internodes in greenhouse conditions, Supplementary Figure S2), it is not as severe as the line WI1983Hde with a complete deletion of *CsTFL1*, and it is not sensitive to length of the day as the lines with a substitution or downregulation of the *CsTFL1d* gene^34–36^.

In early studies, the dominant gene for determinate growth in cucumber was proposed^25^, but later, only recessive genes were reported^26,27,29,34–36,38^. To confirm the recessive character of the *de-2* gene, we performed a genetic analysis, and the results confirmed the recessive character of the *de-2*gene described by Soltysiak et al.^38^. In this study, we report a new variant of *CsTFL1* with a T-to-A substitution in exon 4, which introduces a premature stop codon and a C-terminal deletion of 13 amino acids in CsTFL1 (Y170STOP). Wen et al.^34^. identified a T-to-C substitution in the first exon of *CsTFL1*, resulting in a single amino acid Ser-to-Pro substitution (S71P), explaining the determinate growth of the G421 line, however, it seems that this substitution also exists in indeterminate line 9930 (Fig. 2B). To the best of our knowledge, there are no reports describing premature stop codon mutations in the *TFL1* gene resulting in C-terminal deletion and determinate growth, which makes this type of *TFL1* modification an interesting strategy for modulating plant architecture.

The SAM transition regulation model for cucumber was proposed by Wen et al.^34^. and updated by Wen et al.^35^. In our study, we observed reduced expression of *CsTFL1* in *determinate-2* plants. It is possible, but unlikely, that this explains the phenotype because *CsTFL1* is still expressed in *de-2* plants. Modeling of CsTFL1 showed that C-terminal truncation alters the structure of the protein, possibly disturbing its function. It is possible that the C-terminal deletion of 13 aa in CsTFL1 can influence the direct interaction of this protein with CsNOT2a, influencing the interaction with CsFDP, which is a key interaction for the SAM transition. The interaction of CsTFL1 with the homolog of the miRNA biogenesis protein CsNOT2a was confirmed by Yeast-2-hybrid and Bimolecular Fluorescence Complementation (BiFC) experiments but not investigated in details. It is surprising that the interaction of TFL1 and 14-3-3 was observed in *Arabidopsis*and rice but not in cucumber. Instead Wen et al.^34^ found interaction with NOT2a and FDP. It is possible that different CsTFL1 proteins can form complexes inhibiting expression of the flowering or meristem identity genes, and this would go along with the observation of high variability of segment B in TFL1 proteins. The effects of C-terminal truncations on protein-protein interactions have been reported for other proteins. For example, the impact of C-terminal truncation of the Rab escort protein on the REP-Rab interaction in *Arabidopsis*has been demonstrated^63^. A comparison of morphological traits revealed that the leaves and fruits of the W-sk line were slightly different from those of B10; W-sk plants produced more male flowers (Supplementary Figure S2B), and mature fruits of W-sk were longer but had a lower diameter (Supplementary Figures S1C, D). This finding suggested that CsTFL1 can also affect other morphological traits of cucumber. It has been suggested that TFL1-like proteins interact not only with FD/FDP to regulate SAM transition but also with other transcription factors to modulate plant architecture^24,64^. It seems that not all interactors of CsTFL1 have been identified so far^34,35^. Therefore, further studies of the CsTFL1 interactome would improve the understanding of the function of TFL1 in plants.

In this study, we describe for the first time a chemically-induced variant of CsTFL1, with a substitution in the last exon, resulting in a premature stop codon introduction (Fig. 3). To our knowledge, such a variant of *TFL1* has not been found in previously studied cucumber lines or other plants. This study paves the way for exploring a TFL1 C-terminal truncation strategy to alter the growth of cucumber plants. Functional analysis of the C-terminal region can be applied to design different alleles of *CsTFL1* via gene editing for fine-tuning of the indeterminate/determinate growth habit of cucumber plants to adapt it to modern production systems. The strategy of C-terminal truncation of TFL1 could also be applied to other crop species. Overall, this study provides new resources and novel insights for further advancements to improve plant growth architecture.

## Electronic supplementary material

Below is the link to the electronic supplementary material.

Supplementary Material 1

Supplementary Material 2

## Data Availability

The data generated or analyzed during this study are included in this article and its supplementary files or are available from the corresponding author upon reasonable request.

## References

[1] Bradley, D., Ratcliffe, O., Vincent, C., Carpenter, R. & Coen, E. Inflorescence commitment and architecture in *Arabidopsis*. *Science*. **275**, 80–83 (1997). 8974397 10.1126/science.275.5296.80

[2] Maršić, N. K., Osvald, J. & Jakše, M. Evaluation of ten cultivars of determinate tomato (*Lycopersicum Esculentum* Mill.), grown under different climatic conditions. *Acta Agric. Slov.* **85**, 2:321–328 (2005).

[3] Foley, T. C., Orf, J. H. & Lambert, J. W. Performance of related determinate and indeterminate soybean lines. *Crop Sci.* **26**, 5–8 (1986).

[4] Uzun, B. & Cagirgan, M. I. Comparison of determinate and indeterminate lines of sesame for agronomic traits. *Field Crops Res.* **96**, 13–18 (2006).

[5] Stützel, H. & Aufhammer, W. Grain yield in determinate and indeterminate cultivars of *Vicia faba* with different plant distribution patterns and population densities. *J. Agric. Sci.* **118**, 343–352 (1992).

[6] Naegele, R. P. & Wehner, T. C. Genetic resources of cucumber. In: (eds Grumet, R., Katzir, N. & Garcia-Mas, J.) Genetics and Genomics of Cucurbitaceae. Springer International Publishing AG, Cham, Switzerland, 61–86 (2016).

[7] Eshed, Y. & Lippman, Z. B. Revolutions in agriculture chart a course for targeted breeding of old and new crops. *Science*. **366**, 6466 (2019). 10.1126/science.aax0025 31488704

[8] Wang, S., Yang, Y., Chen, F. & Jiang, J. Functional diversification and molecular mechanisms of *FLOWERING LOCUS T/TERMINAL FLOWER 1* family genes in horticultural plants. *Mol. Hortic.* **16**, 19 (2022). 10.1186/s43897-022-00039-8 PMC10515248 37789396

[9] Chailakhyan, M. K. Internal factors of plant flowering. *Annu. Rev. Plant. Physiol.* **19**, 1–37 (1968).

[10] Kardailsky, I. et al. Activation tagging of the floral inducer FT. *Science*. **286**, 1962–1965 (1999). 10583961 10.1126/science.286.5446.1962

[11] Corbesier, L. et al. FT protein movement contributes to long-distance signaling in floral induction of *Arabidopsis*. *Science*. **316**, 1030–1033 (2007). 17446353 10.1126/science.1141752

[12] Abe, M. et al. FD, a bZIP protein mediating signals from the floral pathway integrator FT at the shoot apex. *Science*. **309**, 1052–1056 (2005). 16099979 10.1126/science.1115983

[13] Bowman, J. L., Alvarez, J., Weigel, D., Meyerowitz, E. M. & Smyth, D. R. Control of flower development in *Arabidopsis thaliana* by and interacting genes. *Development*. **119**, 721–743 (1993).

[14] Liljegren, S. J., Gustafson-Brown, C., Pinyopich, A., Ditta, G. S. & Yanofsky, M. F. Interactions among APETALA1, LEAFY, and TERMINAL FLOWER1 specify meristem fate. *Plant. Cell.* **11**, 1007–1018 (1999). 10368173 10.1105/tpc.11.6.1007 PMC144247

[15] Kobayashi, Y., Kaya, H., Goto, K., Iwabuchi, M. & Araki, T. A pair of related genes with antagonistic roles in mediating flowering signals. *Science*. **286**, 1960–1962 (1999). 10583960 10.1126/science.286.5446.1960

[16] Taoka, K. I. et al. 14-3-3 proteins act as intracellular receptors for rice Hd3a florigen. *Nature*. **476**, 332–335 (2011). 21804566 10.1038/nature10272

[17] Collani, S., Neumann, M., Yant, L. & Schmid, M. FT modulates genome-wide DNA-Binding of the bZIP transcription factor FD. *Plant. Physiol.* **180**, 367–380 (2019). 30770462 10.1104/pp.18.01505 PMC6501114

[18] Fan, L. et al. Comparative genomic analysis of PEBP genes in cucurbits explores the interactors of cucumber CsPEBPs related to flowering time. *Int. J. Mol. Sci.* **25**, 3815 (2024). 38612626 10.3390/ijms25073815 PMC11011414

[19] Bradley, D. et al. Control of inflorescence architecture in *Antirrhinum*. *Nature*. **379**, 791–797 (1996). 8587601 10.1038/379791a0

[20] Ratcliffe, O. J. et al. A common mechanism controls the life cycle and architecture of plants. *Development*. **125**, 1609–1615 (1998). 9521899 10.1242/dev.125.9.1609

[21] Pnueli, L. et al. The SELF-PRUNING gene of tomato regulates vegetative to reproductive switching of sympodial meristems and is the ortholog of CEN and TFL1. *Development*. **125**, 1979–1989 (1998). 9570763 10.1242/dev.125.11.1979

[22] Pnueli, L. et al. Tomato SP-interacting proteins define a conserved signaling system that regulates shoot architecture and flowering. *Plant. Cell.* **13**, 2687–2702 (2001). 11752381 10.1105/tpc.010293 PMC139482

[23] Périlleux, C., Bouché, F., Randoux, M. & Orman-Ligeza, B. Turning meristems into fortresses. *Trends Plant. Sci.* **24**, 431–442 (2019). 30853243 10.1016/j.tplants.2019.02.004

[24] Colleoni, P. E., van Es, S. W., Winkelmolen, T., Immink, R. G. H. & van Esse, G. W. Flowering time genes branching out. *J. Exp. Bot.* **75**, 4195–4209 (2024). 38470076 10.1093/jxb/erae112 PMC11263490

[25] Hutchins, A. E. Inheritance in the cucumber. *J. Agr Res.* **60**, 117–128 (1940).

[26] Ödland, M. L. & Groff, D. W. Linkage of vine type and geotropic response with sex forms in cucumbers *Cucumis sativus* L. *Proc. Amer Soc. Hort Sci.* **82**, 358–369 (1963).

[27] George, W. L. Genetic and environmental modification of determinate plant habit in cucumbers. *J Am Soc Hort Sci.* **95**, 583 (1970).

[28] Miller, G. A. & George, W. L. Inheritance of dwarf and determinate growth habits in cucumber. *J. Am. Soc. Hort Sci.* **104**, 114–117 (1979).

[29] Denna, D. W. Expression of determinate habit in cucumbers (*Cucumis sativus* L). *J. Am. Soc. Hort Sci.* **96**, 277–279 (1971).

[30] Fazio, G., Staub, J. & Stevens, M. Genetic mapping and QTL analysis of horticultural traits in cucumber (*Cucumis sativus* L.) using recombinant inbred lines. *Theor. Appl. Genet.* **107**, 864–874 (2003). 12827247 10.1007/s00122-003-1277-1

[31] Sato, H., Heang, D., Sassa, H. & Koba, T. Identification and characterization of *FT/TFL1* gene family in cucumber. *Breed. Sci.* **59**, 3–11 (2009).

[32] Weng, Y., Johnson, S., Staub, J. E. & Huang, S. An extended intervarietal microsatellite linkage map of cucumber, *Cucumis sativus* L. *Am. Soc. Hortic. Sci.* **45**, 882–886 (2010).

[33] Zhao, W. S., Gu, R., Che, G., Cheng, Z. & Zhang, X. *CsTFL1b* may regulate the flowering time and inflorescence architecture in cucumber (*Cucumis sativus* L). *Biochem. Biophys. Res. Common.* **499**, 307–313 (2018). 10.1016/j.bbrc.2018.03.153 29574158

[34] Wen, C. L. et al. CsTFL1 inhibits determinate growth and terminal flower formation through interaction with CsNOT2a in cucumber. *Development*. **146**, dev180166 (2019). 31320327 10.1242/dev.180166 PMC6679365

[35] Wen, H. et al. TERMINAL FLOWER1 and TERMINAL FLOWER1d respond to temperature and photoperiod signals to inhibit determinate growth in cucumber. *Plant. Cell. Environ.* **44**, 2580–2592 (2021). 33938004 10.1111/pce.14075

[36] Njogu, M. K. et al. A novel mutation in TFL1 homolog sustaining determinate growth in cucumber (*Cucumis sativus* L). *Theor. Appl. Genet.* **133**, 3323–3332 (2020). 32857171 10.1007/s00122-020-03671-4

[37] Ma, C. et al. Comparative analysis of miRNA and mRNA abundance in determinate cucumber by high-throughput sequencing. *PLoS ONE*. **13**, e0190691 (2018). 29304061 10.1371/journal.pone.0190691 PMC5755913

[38] Sołtysiak, U., Kubicki, B. & Korzeniewska, A. Induced mutations in the cucumber (*Cucumis sativus* L.). VI. Determinate type of growth. *Genetica Pol.* **27**, 289–298 (1986).

[39] Kubicki, B. Induced mutations on cucumber (*Cucumis sativus* L.) I. variability in M1 and M2 generations. *Genet. Pol.* **24**, 343–353 (1983).

[40] Słomnicka, R. et al. Genetic mapping of *psl* locus and quantitative trait loci for angular leaf spot resistance in cucumber (*Cucumis sativus* L). *Mol. Breed.* **38**, 111 (2018). 30174539 10.1007/s11032-018-0866-2 PMC6105252

[41] Osipowski, P. et al. A high-quality cucumber genome assembly enhances computational comparative genomics. *Mol. Genet. Genom*. **295**, 177–193 (2020). 10.1007/s00438-019-01614-3 31620884

[42] Li, H. & Durbin, R. Fast and accurate short read alignment with Burrows–Wheeler transform. *Bioinformatics*. **25**, 1754–1760 (2009). 19451168 10.1093/bioinformatics/btp324 PMC2705234

[43] Li, H. et al. The sequence Alignment/Map format and SAMtools. *Bioinformatics*. **25**, 2078–2079 (2009). 19505943 10.1093/bioinformatics/btp352 PMC2723002

[44] Yu, J. et al. CuGenDBv2 an updated database for cucurbit genomics. *Nucleic Acids Res.* **51** (D1), D1457–D1464 (2023). 36271794 10.1093/nar/gkac921 PMC9825510

[45] Mróz, T. L., Havey, M. J. & Bartoszewski, G. Cucumber possesses a single terminal alternative oxidase gene that is upregulated by cold stress and in the mosaic (MSC) mitochondrial mutants. *Plant. Mol. Biol. Rep.* **33**, 1893–1906 (2015). 10.1007/s11105-015-0883-9 PMC4695503 26752808

[46] Livak, K. J. & Schmittgen, T. D. Analysis of relative gene expression data using real-time quantitative PCR and the 2^–∆∆Ct^ method. *Methods*. **25**, 402–408 (2001). 11846609 10.1006/meth.2001.1262

[47] Robinson, J. T. et al. Integrative genomics viewer. *Nat. Biotechnol.* **29**, 24–26 (2011). 21221095 10.1038/nbt.1754 PMC3346182

[48] Madeira, F. et al. The EMBL-EBI Job dispatcher sequence analysis tools framework in 2024. *Nucleic Acids Res.* gkae241. 10.1093/nar/gkac241 (2024). 10.1093/nar/gkae241 PMC11223882 38597606

[49] UniProt Consortium, T. UniProt: the universal protein knowledgebase. *Nucleic Acids Res.* **46**, 2699 (2018). 29425356 10.1093/nar/gky092 PMC5861450

[50] Benson, D. A. et al. GenBank. *Nucleic Acids Res.* **41**, D36–42 (2013). 23193287 10.1093/nar/gks1195 PMC3531190

[51] Serre, L., Vallée, B., Bureaud, N., Schoentgen, F. & Zelwer, C. Crystal structure of the phosphatidylethanolamine-binding protein from bovine brain: a novel structural class of phospholipid-binding proteins. *Structure*. **6**, 1255–1265 (1998). 9782057 10.1016/s0969-2126(98)00126-9

[52] Ahn, J. H. et al. A divergent external loop confers antagonistic activity on floral regulators FT and TFL1. *EMBO J.* **253**, 605–614 (2006). 10.1038/sj.emboj.7600950 PMC1383534 16424903

[53] Mirdita, M. et al. ColabFold: making protein folding accessible to all. *Nat. Methods*. **19**, 679–682 (2022). 35637307 10.1038/s41592-022-01488-1 PMC9184281

[54] Schrödinger, L. L. C. The PyMOL Molecular Graphics System, Version 3.0. accessed from (2024). https://www.pymol.org/

[55] Wang, X. et al. The USDA cucumber (*Cucumis sativus* L.) collection: genetic diversity, population structure, genome-wide association studies and core collection development. *Hortic. Res.* **5**, 64 (2018). 30302260 10.1038/s41438-018-0080-8 PMC6165849

[56] Banfield, M. J. & Brady, R. L. The structure of *Antirrhinum* CENTRORADIALIS protein (CEN) suggests a role as a kinase regulator. *J. Mol. Biol.* **297**, 1159–1170 (2000). 10764580 10.1006/jmbi.2000.3619

[57] Hanzawa, Y., Money, T. & Bradley, D. A single amino acid converts a repressor to an activator of flowering. *Proc. Natl. Acad. Sci. USA*. **102**, 7748–7753 (2005). 15894619 10.1073/pnas.0500932102 PMC1140427

[58] Stevens, M. A. & Rick, C. M. Genetics and breeding. In: (eds Atherton, J. & Rudich, J.) The Tomato crop: A Scientific Basis for Improvement, Chapman and Hall, London, 35–100 (1986).

[59] Li, S. et al. Parallel domestication with a broad mutational spectrum of determinate stem growth habit in leguminous crops. *Plant. J.* **96**, 761–771 (2018). 30112860 10.1111/tpj.14066

[60] Li, K. et al. A novel locus (*Bnsdt2*) in a TFL1 homologue sustaining determinate growth in *Brassica napus*. *BMC Plant. Biol.* **21**, 568 (2021). 34861823 10.1186/s12870-021-03348-0 PMC8641158

[61] Liu, X. F., Chen, J. C. & Zhang, X. L. Genetic regulation of shoot architecture in cucumber. *Hortic. Res.* **8**, 143 (2021). 34193859 10.1038/s41438-021-00577-0 PMC8245548

[62] Crane, M., Wehner, T. C. & Naegele, R. P. Cucumber cultivars for container gardening and the value of field trials for predicting cucumber performance in containers. *HortScience*. **53**, 16–22 (2018).

[63] Gutkowska, M. et al. Impact of C-terminal truncations in the *Arabidopsis* Rab escort protein (REP) on REP-Rab interaction and plant fertility. *Plant. J.* **108**, 1400–1421 (2021). 34592024 10.1111/tpj.15519 PMC9293207

[64] Cerise, M. et al. Two modes of gene regulation by TFL1 mediate its dual function in flowering time and shoot determinacy of *Arabidopsis*. *Development*. **150**, dev202089 (2023). 37971083 10.1242/dev.202089 PMC10730086

