# Supplementary material for: A single nucleotide substitution introducing premature stop codon within CsTFL1 explains the determinate-2 phenotype in cucumber (Cucumis sativus L.)
Source: Sci Rep. 2024 Oct 25;14:25368. doi: 10.1038/s41598-024-76549-w (PMC11511849; doi:10.1038/s41598-024-76549-w)
Supplement: Supplementary file 1 — Supplementary Material 1 [file 41598_2024_76549_MOESM1_ESM.docx]

**Supplementary Figures**

**Supplementary Figure S1A**

**
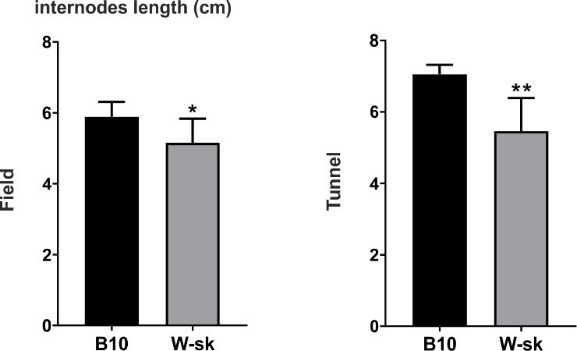
**

Comparison of internode length in W-sk (*de-2*) and B10 (WT) 8-week-old plants grown in the field and tunnel. For each line, three independent biological replicates were used, with a minimum of three plants per line in each biological replicate. The error bars represent the SDs, and the asterisks indicate significant differences according to the Student’s t-test: **P* < 0.05 and ***P* < 0.01.

**Supplementary Figure S1B**


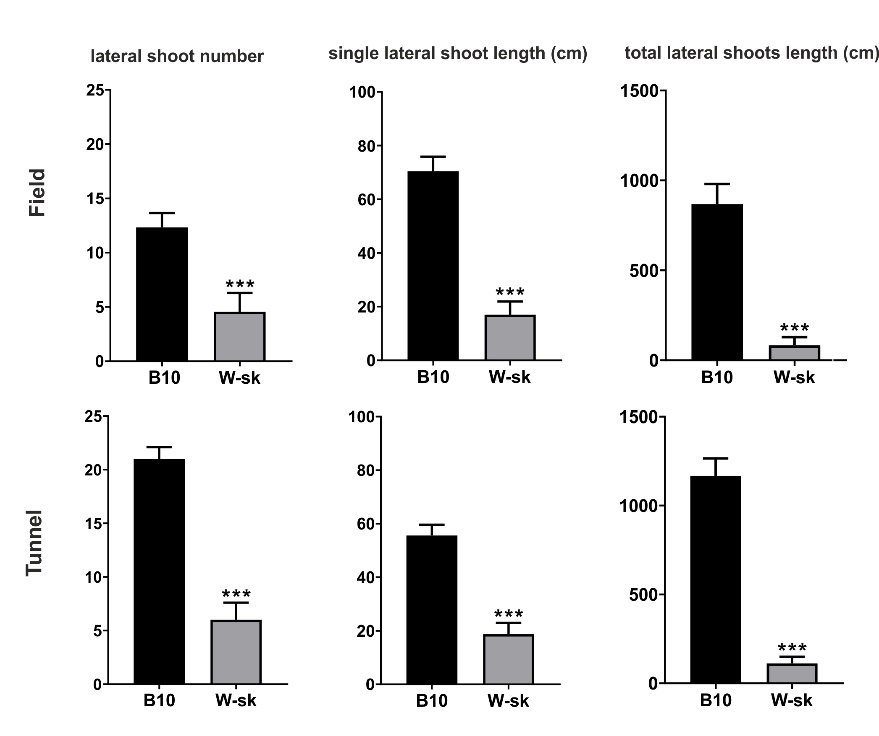


Comparison of lateral shoots in W-sk (*de-2*) and B10 (WT) plants collected from 8-week-old plants grown in the field and tunnel. For each line, three independent biological replicates were used, with a minimum of three plants per line in each biological replicate. The error bars represent the SDs, and the asterisks indicate significant differences according to the Student’s t-test; ****P* < 0.001.

**Supplementary Figure S1C**


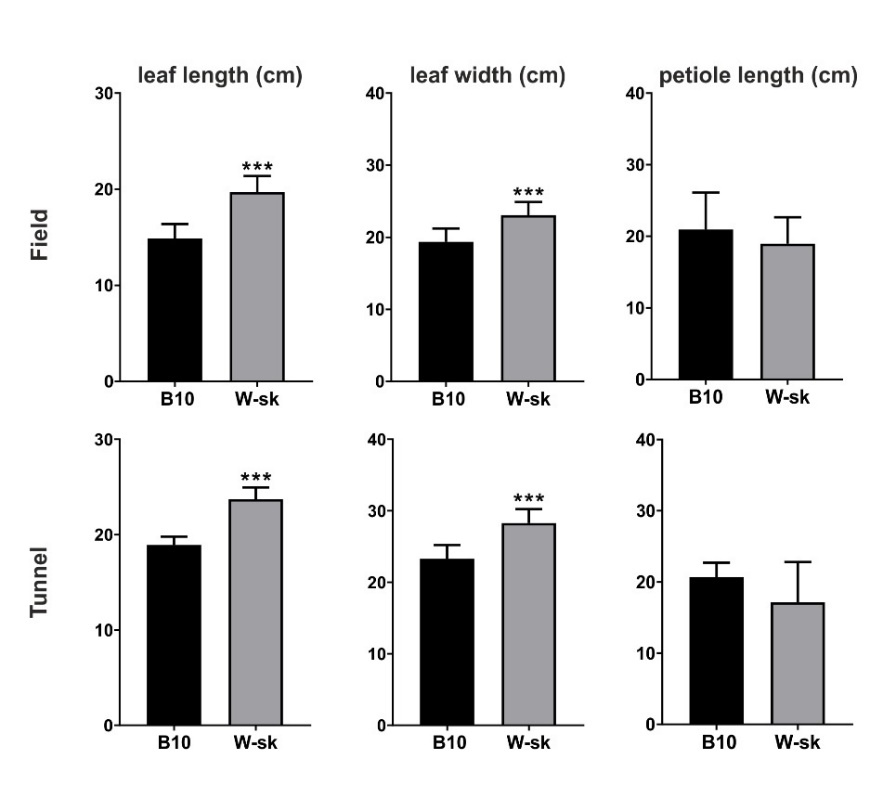


Comparison of fully developed leaves in W-sk (*de-2*) and B10 (WT) plants collected from 8-week-old plants grown in the field and tunnel. For each line, three independent biological replicates were used, with a minimum of three leaves per line in each biological replicate. The error bars represent the SDs, and the asterisks indicate significant differences according to the Student’s t-test; ****P* < 0.001.

**Supplementary Figure S1D**

**
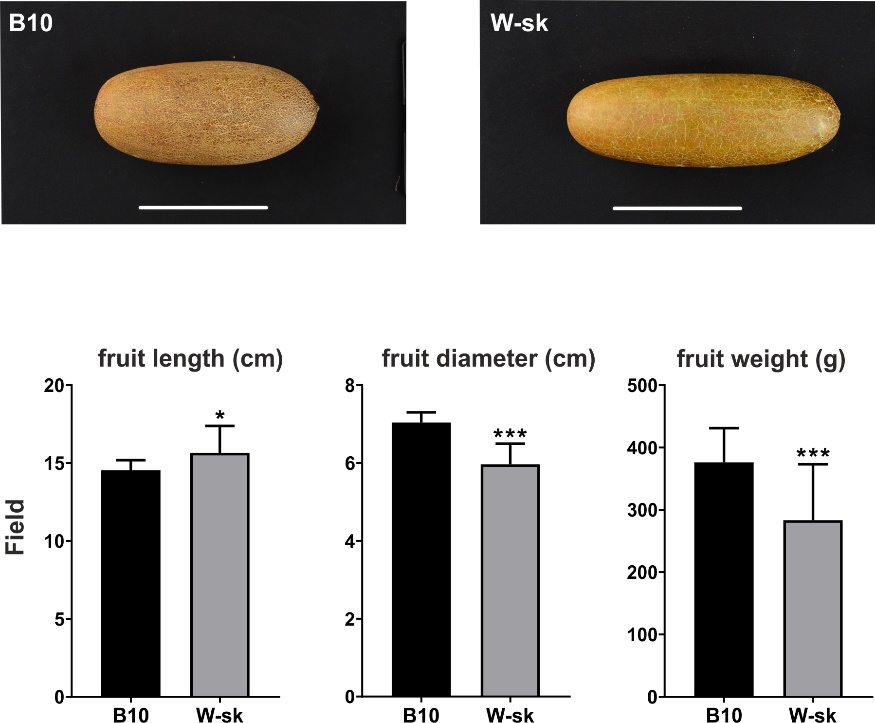
**

Comparison of mature fruits of W-sk (*de-2*) and B10 (WT) plants harvested from the field. For each line, three independent biological replicates were used, and a minimum of three fruits per line were measured. The line bars represent 10 cm. The error bars represent the SDs, and the asterisks indicate significant differences according to the Student’s t-test: * *P* < 0.05 and ****P* < 0.001.

**Supplementary Figure S2**


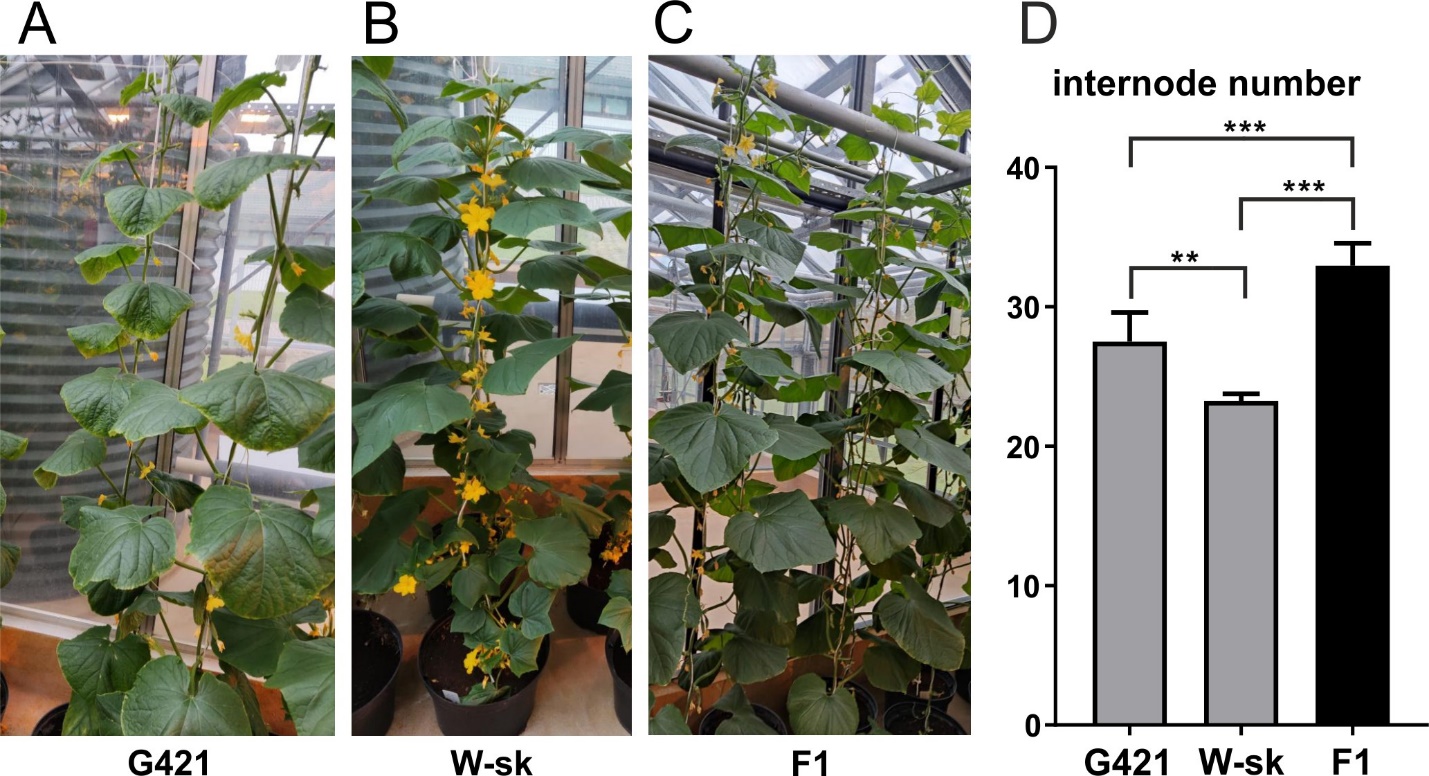


The photographs of representative plants of parental lines G421, B10, and F1 used in the allelism test (A-C), and a plot presenting an average internode number for each line and F1 (D). The data are presented as the means ± SDs of internode number for four plants of each parental line and 40 F1 plants. Student’s t-test; ** *P* < 0.01 and *** *P* < 0.001.
